# Effects of Sintering Parameters on the Microstructure and Optical Transmittance of Monolithic 4 mol% Yttria-Partially Stabilized Zirconia

**DOI:** 10.3390/bioengineering13060702

**Published:** 2026-06-19

**Authors:** Taek-Jun Chung, Myung-Joo Kim, Ho-Beom Kwon, Bongju Kim, Young-Jun Lim

**Affiliations:** 1 Department of Prosthodontics and Dental Research Institute, School of Dentistry, Seoul National University, Seoul 03085, Republic of Korea; taekjunn@snu.ac.kr (T.-J.C.); silk1@snu.ac.kr (M.-J.K.); proskwon@snu.ac.kr (H.-B.K.); 2 Dental Life Science Research Institute, Seoul National University Dental Hospital, Seoul 03080, Republic of Korea; bjkim016@snu.ac.kr

**Keywords:** dental materials, electron microscopy, sintering, spectrophotometry, zirconium oxide, 4Y-PSZ, optical transmittance

## Abstract

High-translucency 4 mol% yttria-partially stabilized zirconia (4Y-PSZ) is widely used for esthetic restorations, but sintering conditions that balance translucency and microstructural control remain unclear. This study evaluated the independent effects of peak temperature, holding time, and heating rate on the microstructure and total luminous transmittance of monolithic 4Y-PSZ. Disks were sintered at peak temperatures of 1470–1560 °C, holding times of 30–180 min, and heating rates of 3–10 °C/min. Grain size and internal defect density (≥0.5 µm) were quantified by scanning electron microscopy, and total luminous transmittance at 0.5 mm thickness was measured using a spectrophotometer. Higher peak temperatures and longer holding times increased grain size (0.481 ± 0.020 to 0.785 ± 0.035 µm, and 0.503 ± 0.037 to 0.730 ± 0.041 µm, respectively) and reduced defect density, whereas heating rate had no significant effect on either. Transmittance remained within a narrow range (approximately 40–43% at 0.5 mm) across all schedules, with 1560 °C yielding the lowest value. These findings indicate that the microstructure of monolithic 4Y-PSZ is governed primarily by peak temperature and holding time, while transmittance is relatively insensitive to the sintering schedule. Practically, a peak temperature of 1500–1530 °C with a 1–2 h hold provides a robust processing window balancing densification, grain coarsening, and optical performance for clinical workflows.

## 1. Introduction

Monolithic zirconia has expanded from posterior full-contour crowns to anterior esthetic restorations because of its high strength, biocompatibility, and compatibility with CAD/CAM systems [1,2,3]. However, a fundamental trade-off between optical esthetics and mechanical reliability remains in both single-unit and multi-unit prostheses [4,5]. Although high-translucency formulations containing 4–5 mol% Y_2_O_3_ improve optical outcomes compared with conventional 3 mol% yttria-stabilized tetragonal zirconia polycrystal (3Y-TZP), concerns remain regarding strength, fracture behavior, and low-temperature degradation (LTD) [6,7,8,9]. A recent systematic review and meta-analysis identified yttria content, specimen thickness, and sintering conditions as the principal material-related variables influencing the translucency of contemporary zirconia [10].

The optical properties of zirconia arise from the interplay of several microstructural factors, including phase assemblage, grain size, porosity, secondary phases such as alumina, and processing-induced defects [1,11,12]. Light transmission through polycrystalline zirconia is governed primarily by two scattering mechanisms: birefringent scattering at grain boundaries between anisotropic tetragonal grains and scattering at residual pores and other internal defects. Increasing the cubic fraction reduces birefringent scattering and improves light transmission, but simultaneously diminishes transformation toughening; this reflects the well-recognized trade-off in which higher translucency is generally accompanied by lower strength [1]. Consequently, optimizing the optical performance of a given zirconia composition is not a matter of maximizing any single parameter, but of balancing competing microstructural contributions–a complexity that motivates the systematic, parameter-by-parameter approach adopted in this study.

At comparable density and phase composition, coarser grains in tetragonal-rich zirconia increase birefringence-related scattering at grain boundaries and thereby reduce translucency [12]. Thus, apparent increases in translucency at higher sintering temperatures usually reflect pore elimination and/or an increased cubic fraction rather than a direct benefit of larger grains [5,12,13,14,15].

Sintering parameters, including peak temperature, holding time, and heating rate, govern densification, grain growth, and pore elimination or retention; consequently, the optical and mechanical properties of the final ceramic depend on the firing schedule [5,13,15,16,17].

Previous studies of speed and high-speed sintering have reported inconsistent findings. Some studies have reported optical and mechanical properties comparable between speed-sintered and conventionally sintered yttria-stabilized zirconia [5,18,19,20], whereas others have reported reductions in flexural strength, light transmittance, or the CIELab-based translucency parameter after speed-sintering protocols [21,22,23]. However, many speed-sintering protocols alter peak temperature, holding time, and heating rate simultaneously, making it difficult to attribute observed changes to any single parameter. Heating rate, in particular, remains understudied as an independent variable. Although two studies independently isolated heating rate as a sintering variable in monolithic zirconia, both focused on 3Y-TZP and evaluated flexural strength only, without assessing microstructure or optical properties [24,25]. Thus, the independent effect of heating rate on both microstructure and optical properties remains unestablished for 4Y-PSZ specifically [7].

In addition, prior studies have focused predominantly on 3Y-TZP, with comparatively few investigations evaluating 4Y-PSZ in isolation. Furthermore, internal defect density, defined as the area-normalized count of pore-like features ≥0.5 μm, has not been reported as an independent outcome, as most studies rely on representative scanning electron microscopy (SEM) micrographs without quantitative analysis. Therefore, the quantitative relationship between sintering-parameter-driven changes in defect density and total luminous transmittance in 4Y-PSZ remains unclear. Accordingly, this study independently evaluated the effects of peak sintering temperature, holding time, and heating rate on grain size, area-normalized internal defect density (≥0.5 µm), and total luminous transmittance in single-shade monolithic 4Y-PSZ. The null hypotheses were that sintering temperature, holding time, and heating rate would have no significant effect on grain size, area-normalized defect density (≥0.5 µm), or total luminous transmittance in monolithic single-shade 4Y-PSZ.

## 2. Materials and Methods

### 2.1. Materials and Specimen Preparation

Commercial 4 mol% yttria-partially stabilized zirconia blocks (4Y-PSZ; Estar-Z HT, shade A2; Osstem Implant Co., Ltd., Seoul, Republic of Korea) were used in this study. According to the manufacturer, the blocks were produced from zirconia powder supplied by Tosoh Corporation (Tokyo, Japan) and had a monolithic single-shade A2 composition without a layered structure. Pre-sintered disk-shaped specimens were sectioned from the blocks using a precision cutting machine (IsoMet 4000; Buehler Ltd., Lake Bluff, IL, USA), ground plane-parallel using a grinding/polishing system (MetPrep 3; Allied High Tech Products, Inc., Cerritos, CA, USA), and mirror-polished with a 1-μm diamond suspension [15]. Green-body dimensions were calculated to yield sintered disk-shaped specimens with a final diameter of 13 mm and a final thickness of 0.5 mm. For each of the 12 experimental groups, three specimens were allocated for transmittance measurement and three for scanning electron microscopy (SEM)-based microstructural analysis, yielding *n* = 3 per outcome per group. The nominal reference condition (1530 °C/120 min/5 °C/min) was included independently in all three parameter series (Tp1530, Tm120, and HR5), and each reference group was prepared in a separate furnace run to serve as a within-series control. Thus, the total specimen count was *N* = 72 (12 groups × 3 specimens × 2 outcomes).

This material was selected because it is widely used in clinical practice and is manufactured from zirconia powder supplied by Tosoh Corporation, which also serves as the raw material basis for several other major commercial 4Y-PSZ products, including Katana Zirconia HT (Kuraray Noritake Dental Inc., Tokyo, Japan), Ceramill Zolid HT+ (Amann Girrbach AG, Koblach, Austria), and IPS e.max ZirCAD MT (Ivoclar Vivadent AG, Schaan, Liechtenstein) [5]. Although compositional differences introduced during downstream manufacturing cannot be excluded, this shared powder origin may support cautious comparison with other 4Y-PSZ materials.

### 2.2. Sintering Protocols

All specimens were sintered in a high-temperature furnace (S-6100; Addin Co., Ltd., Goyang-si, Gyeonggi-do, Republic of Korea) with a maximum operating temperature of 1600 °C. The furnace accommodated up to two trays, with 25 specimens per tray, and had a chamber volume of 110 × 110 × 150 mm. The 12 experimental groups were classified into three sintering-parameter series. The fixed parameters in each series corresponded to the manufacturer’s recommended sintering protocol for this material: peak temperature, 1530 °C; heating rate, 5 °C/min; and holding time, 120 min. Thus, each variable was tested against a clinically relevant reference condition.

- Peak-temperature series (Tp1470, Tp1500, Tp1530, and Tp1560): sintering temperatures of 1470, 1500, 1530, and 1560 °C, with a heating rate of 5 °C/min and a holding time of 120 min.
- Holding-time series (Tm30, Tm60, Tm120, and Tm180): holding times of 30, 60, 120, and 180 min, with a peak temperature of 1530 °C and a heating rate of 5 °C/min.
- Heating-rate series (HR3, HR5, HR7, and HR10): heating rates of 3, 5, 7, and 10 °C/min, with a peak temperature of 1530 °C and a holding time of 120 min.

After sintering, all specimens were allowed to cool naturally to room temperature inside the closed furnace chamber (Table 1).

### 2.3. Microstructural Characterization by Scanning Electron Microscopy

Microstructural features, including grain size and defect density, were analyzed using field-emission scanning electron microscopy (JSM-7610F Plus; JEOL Ltd., Tokyo, Japan; resolution, 0.8 nm at 15 kV). Before SEM observation, sintered specimens were wet-polished with a 1 µm diamond suspension and thermally etched at 1400 °C for 60 min, with a heating rate of 10 °C/min and natural cooling inside the furnace. A platinum conductive layer was applied by sputter coating at 10 mA for 150 s in three consecutive cycles to ensure a continuous coating.

For grain-size analysis, SEM images were captured at ×10,000 magnification, an accelerating voltage of 15 kV, and a working distance of 8 mm. For each group, three specimens were examined, with three to four randomly selected non-overlapping fields per specimen, totaling 10 fields per group and more than 100 grains per group. Grain size was determined using the linear-intercept method in accordance with ASTM E112-24 [26] and mean grain size (d) was calculated as d = 1.56 × L^−^, where L^−^ is the mean linear intercept, in accordance with Mendelson’s correction for polycrystalline ceramics [27]. Group means and standard deviations were then computed from these 10 field values.

Defect density was evaluated using SEM images acquired at ×1000 magnification. For each group, 30 fields were obtained from three specimens, with 10 fields per specimen; observation sites were randomly selected and spaced at least 1 mm apart on the specimen surface to improve representativeness. Pore-like dark features with an equivalent diameter of ≥0.5 µm were identified and counted in ImageJ software (version 1.54; National Institutes of Health, Bethesda, MD, USA) by a single calibrated examiner after training with a set of 20 reference images. To assess intraobserver reliability, a random 10% subset of all fields was re-evaluated by the same examiner after a minimum two-week interval; intraobserver agreement was excellent (ICC = 0.93; 95% CI, 0.88–0.96), based on a two-way mixed-effects, absolute-agreement, single-measurement model. For quantitative comparisons among groups, defect density was defined as the total number of identified defects per total analyzed area (field-of-view area × number of fields per group) and expressed as counts·mm^−2^.

The threshold of ≥0.5 μm was chosen for three reasons. First, at the acquired magnification (×1000) and a pixel size of approximately 0.094 μm/pixel (scale bar: 10 μm = 107 pixels), a 0.5-μm defect corresponds to approximately 22 pixels^2^, which is well above the practical detection limit of ≥3 × 3 pixels required for reliable discrimination from sub-pixel noise. Second, at this threshold, pore-like features showed a characteristic dark central core surrounded by a bright edge-enhancement rim in secondary-electron imaging, enabling reliable discrimination from grain-boundary triple junctions. Third, pores with diameters comparable to or greater than the wavelength of visible light (λ ≈ 0.4–0.7 μm; Mie-scattering regime) are principal scattering centers governing the total luminous transmittance of translucent zirconia, and a 0.5-μm threshold captures the lower end of this regime [12,15].

### 2.4. Optical Transmittance Measurement

Total luminous transmittance was measured using a benchtop spectrophotometer (CM-5; Konica Minolta, Inc., Tokyo, Japan) in accordance with ISO 13468-1:2019 [28]. For each group, three disk-shaped specimens, 13 mm in diameter and 0.5 mm in nominal thickness, were finished with double-sided mirror polishing using a 1-μm diamond suspension. After polishing, the thickness of each specimen was verified with a digital micrometer (resolution 0.001 mm), and all specimens were within 0.50 ± 0.02 mm (i.e., a maximum deviation of ±0.02 mm from the nominal value). Measurements were performed under the CIE standard illuminant D65 with a 10° observer. For each specimen, three repeated measurements were obtained and averaged to yield a single representative transmittance value per specimen, which was then used as the unit of analysis (*n* = 3 per group).

### 2.5. Statistical Analysis

All statistical analyses were performed using IBM SPSS Statistics for macOS (version 29.0.2.0; IBM Corp., Armonk, NY, USA), and the significance level was set at α = 0.05 for all two-tailed tests.

Normality and homogeneity of variance were assessed using the Shapiro–Wilk test and Levene’s test, respectively. Grain size was analyzed separately within each parameter series using one-way analysis of variance (ANOVA) when both normality and homogeneity of variance were satisfied or Welch’s ANOVA when homogeneity of variance was violated, followed by Tukey’s HSD or Games–Howell post hoc tests, respectively.

Defect density (counts·mm^−2^) was compared within each series using a generalized linear model with a Poisson distribution, log link, and log-transformed analyzed area as an offset. Overdispersion was assessed using the ratio of the Pearson χ^2^ statistic to the residual degrees of freedom; when overdispersion was detected, defined as a ratio >1.5, a negative binomial model was fitted instead. Group effects were evaluated using the likelihood-ratio test (LRT) and Wald test, and pairwise contrasts were expressed as incidence rate ratios (IRRs) with 95% confidence intervals (CIs).

Total luminous transmittance (%) was analyzed within each series using the Kruskal–Wallis test because the small sample size (*n* = 3 per group) precluded robust parametric assumptions; pairwise comparisons were performed using Dunn’s test with Bonferroni correction.

## 3. Results

### 3.1. Grain Size

#### 3.1.1. Peak Temperature

**Figure 1:**
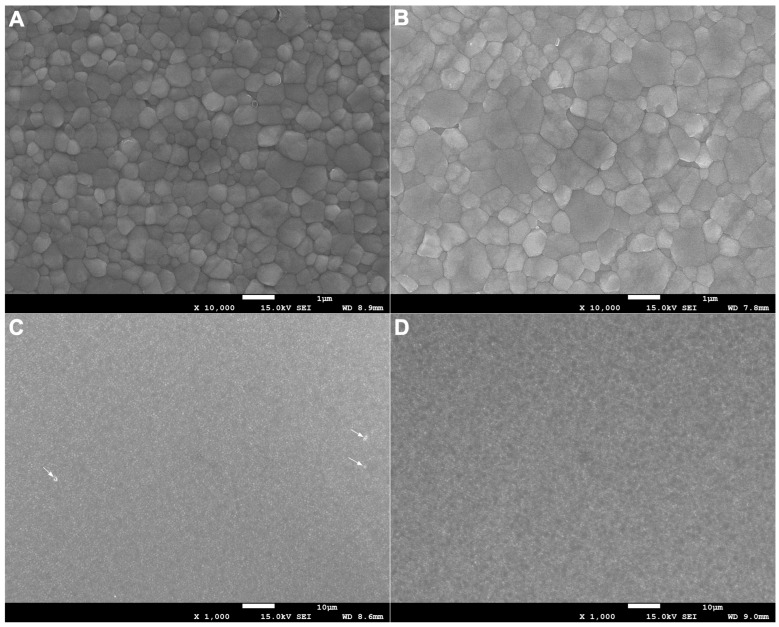
Representative scanning electron micrographs of monolithic 4Y-PSZ sintered at the lowest peak temperature (Tp1470, 1470 °C) and highest peak temperature (Tp1560, 1560 °C), with a holding time of 120 min and heating rate of 5 °C/min. (**A**) Tp1470 at ×10,000 magnification showing fine equiaxed grains (mean grain size, 0.48 μm). (**B**) Tp1560 at ×10,000 magnification showing coarser grains (mean grain size, 0.79 μm). (**C**) Tp1470 at ×1000 magnification showing representative pore-like defects ≥0.5 μm (arrows). (**D**) Tp1560 at ×1000 magnification showing no detectable pore-like defects ≥0.5 μm in the displayed field. All specimens were thermally etched at 1400 °C for 60 min prior to imaging. Scale bars = 1 μm in (**A**,**B**) and 10 μm in (**C**,**D**).

**Figure 2:**
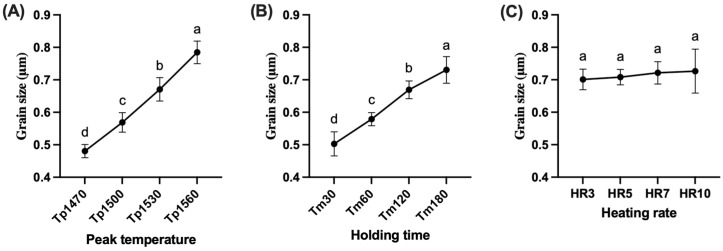
Effects of sintering parameters on the mean grain size of 4Y-PSZ. (**A**) Peak-temperature series: 1470, 1500, 1530, and 1560 °C, with a holding time of 120 min and a heating rate of 5 °C/min. (**B**) Holding-time series: 30, 60, 120, and 180 min, with a peak temperature of 1530 °C and a heating rate of 5 °C/min. (**C**) Heating-rate series: 3, 5, 7, and 10 °C/min, with a peak temperature of 1530 °C and holding time of 120 min. Data are shown as group means ± standard deviation (SD) (*n* = 10 fields per group; ≥100 grains per group). Different lowercase letters above data points indicate significant differences within a series (one-way ANOVA followed by Tukey’s HSD test or Welch’s ANOVA followed by the Games–Howell test; *p* < 0.05).

Mean grain size increased progressively with peak temperature, from 0.481 ± 0.020 μm at 1470 °C to 0.785 ± 0.035 μm at 1560 °C (Figure 1A,B and Figure 2A; Table 2). All pairwise comparisons were significant, indicating a monotonic increase in grain size across the peak-temperature series (Figure 2A; Table 2).

#### 3.1.2. Holding Time

Mean grain size also increased with longer holding time, from 0.503 ± 0.037 μm at 30 min to 0.730 ± 0.041 μm at 180 min (Figure 2B; Table 2). All pairwise comparisons were significant within the holding-time series (Figure 2B; Table 2).

#### 3.1.3. Heating Rate

Mean grain size remained similar across the heating-rate series, ranging from 0.701 ± 0.032 μm at 3 °C/min to 0.727 ± 0.068 μm at 10 °C/min (Figure 2C; Table 2). No significant differences in grain size were detected among heating-rate groups (Figure 2C; Table 2).

### 3.2. Defect Density

#### 3.2.1. Peak Temperature

**Figure 3:**
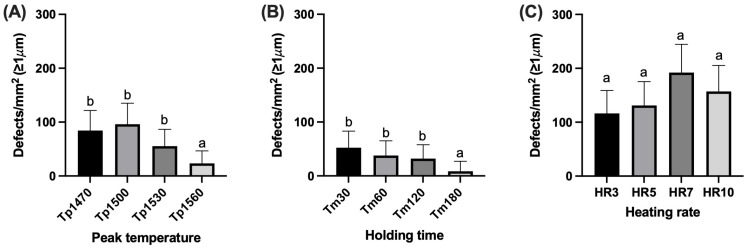
Area-normalized internal defect density, defined as defects ≥0.5 μm, under different sintering schedules. (**A**) Peak-temperature series: 1470, 1500, 1530, and 1560 °C, with a holding time of 120 min and a heating rate of 5 °C/min. (**B**) Holding-time series: 30, 60, 120, and 180 min, with a peak temperature of 1530 °C and a heating rate of 5 °C/min. (**C**) Heating-rate series: 3, 5, 7, and 10 °C/min, with a peak temperature of 1530 °C and holding time of 120 min. Bars represent model-predicted mean defect densities (counts·mm^−2^) from a Poisson generalized linear model with log-transformed analyzed area as an offset; error bars denote 95% confidence intervals (*n* = 30 fields per group). Different lowercase letters indicate significant pairwise differences within a series (*p* < 0.05); in the heating-rate series, the shared superscript ‘a’ indicates no significant differences among groups. Lower values indicate more complete densification.

Intraobserver reliability for defect counting was excellent (ICC = 0.93; 95% CI, 0.88–0.96). Defect density differed across the peak-temperature series (Figure 3A; Table 3). The model-predicted mean defect density was lowest at 1560 °C (Figure 1C,D and Figure 3A; Table 3).

#### 3.2.2. Holding Time

Defect density decreased with longer holding time and was lowest after the 180 min hold (Figure 3B; Table 3). Only the 180 min hold showed a clear reduction in defect density relative to the shorter holding-time groups (Figure 3B; Table 3).

#### 3.2.3. Heating Rate

No significant differences in defect density were detected among heating rate groups (Figure 3C; Table 3).

### 3.3. Optical Transmittance

#### 3.3.1. Peak Temperature

**Figure 4:**
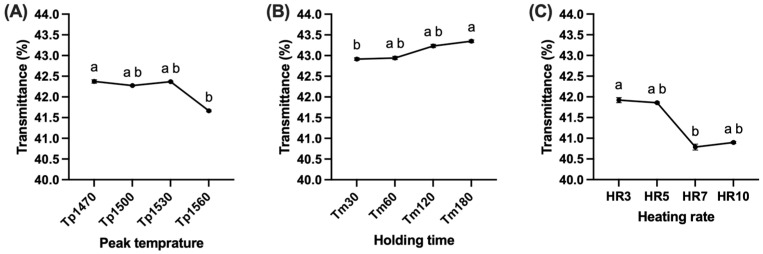
Total luminous transmittance of 0.5 mm-thick 4Y-PSZ disks after different sintering conditions, measured using a spectrophotometer under CIE standard illuminant D65 in accordance with ISO 13468-1:2019. (**A**) Peak-temperature series. (**B**) Holding-time series at 1530 °C. (**C**) Heating-rate series at 1530 °C with a holding time of 120 min. Data are shown as group means ± SD (*n* = 3 specimens per group, with each specimen measured three times). Different lowercase letters indicate significant pairwise differences within a series (Kruskal–Wallis test followed by Dunn’s test with Bonferroni correction; *p* < 0.05). Note the small absolute differences in transmittance and the restricted y-axis range.

Total luminous transmittance remained within a narrow range across the peak-temperature series, although the 1560 °C group showed the lowest value (Figure 4A; Table 4). The only significant pairwise difference was lower transmittance at Tp1560 than at Tp1470 (Figure 4A; Table 4).

#### 3.3.2. Holding Time

Total luminous transmittance increased slightly with longer holding time, from 42.92 ± 0.03% at 30 min to 43.35 ± 0.03% at 180 min (Figure 4B; Table 4). The only significant pairwise difference was higher transmittance at Tm180 than at Tm30 (Figure 4B; Table 4).

#### 3.3.3. Heating Rate

Total luminous transmittance varied only modestly across the heating-rate series, with lower values observed at HR7 and HR10 than at HR3 and HR5 (Figure 4C; Table 4). The only significant pairwise difference was lower transmittance at HR7 than at HR3 (Figure 4C; Table 4).

## 4. Discussion

This study showed that sintering parameters influenced the microstructure and, to a lesser extent, the total luminous transmittance of monolithic 4Y-PSZ. Higher peak temperatures and longer holding times increased grain size and reduced the density of internal defects ≥0.5 μm, whereas heating rate had no significant effect on grain size and no definitive overall effect on defect density within the tested range of 3–10 °C/min. Despite these microstructural changes, total luminous transmittance at 0.5 mm thickness differed only modestly among sintering schedules, consistent with the view that translucency in 4Y-PSZ reflects the combined effects of densification, grain size, and phase composition [5,12,15].

### 4.1. Effect of Sintering Temperature

Increasing the peak sintering temperature increased grain size and reduced defect density. This finding is consistent with thermally activated grain growth and improved densification at higher temperatures. Previous studies on 3Y- and 4Y-based zirconia have likewise reported increased grain size with higher sintering temperatures [13,14,15,16,17]. The reduction in area-normalized defects at higher temperatures suggests more complete pore elimination, which may favor optical clarity because residual pores scatter light [5,12,15]. However, these microstructural changes did not translate into a monotonic increase in translucency. Total transmittance declined slightly at 1560 °C compared with the 1470–1530 °C groups. This finding is consistent with reports that sintering beyond an optimal temperature may adversely affect optical properties [13,14]. Stawarczyk et al. reported that excessive sintering temperatures in 3Y-TZP were associated with microstructural defects and reduced flexural strength, leading them to recommend avoiding temperatures above 1550 °C for that material [13].

In the present 4Y-PSZ, the slight decrease in transmittance at 1560 °C may be attributable to excessive grain growth and increased light scattering, particularly in retained tetragonal regions [12]. Minor changes in phase assemblage or yttrium redistribution may also have contributed, although these mechanisms were not directly assessed. Within the tested range, higher-temperature sintering improved densification but appeared to reach a threshold beyond which translucency no longer improved [13,14,15]. Thus, a peak temperature of approximately 1500–1530 °C appeared to balance densification and grain coarsening in this material.

### 4.2. Effect of Holding Time

Prolonging the dwell at 1530 °C increased grain size and reduced predicted defect density. This behavior reflects the two concurrent, diffusion-controlled processes that operate during isothermal sintering. In the final stage of sintering, extended high-temperature exposure provides additional time for vacancy diffusion and grain-boundary migration, which simultaneously drives the elimination of residual closed pores and promotes grain growth [15,16]. The reduction in defect density after the 180 min hold therefore indicates more complete pore removal, whereas the accompanying increase in grain size reflects the coarsening that accompanies prolonged isothermal exposure, consistent with the grain-growth kinetics described for yttria-stabilized tetragonal zirconia [17]. These two processes have opposing optical consequences: residual porosity is a dominant source of light scattering because of the large reflective-index mismatch between pores and the zirconia matrix, so its removal increases transmittance, whereas coarser grains increase birefringent scattering at tetragonal grain boundaries and tend to reduce transmittance [5,12,15]. The slight net increase in transmittance after the 180 min hold suggests that, in this material, the optical benefit of pore elimination modestly outweighed the penalty from grain coarsening within the tested dwell range. Consistent with this interpretation, total luminous transmittance was slightly higher after the 180 min hold than after the 30 min hold. Longer firing times therefore appeared to allow more complete densification, with only a small optical gain. However, the transmittance gains were modest, suggesting diminishing optical returns once near-full density is approached. This finding aligns with prior evidence that holding time can be shortened without substantial optical compromise when near-full density is attained [7,23].

In the present study, even the 30 min hold produced transmittance close to that of the 120 min reference condition, suggesting that shorter holding times may be feasible for this material. However, prolonged sintering also promoted grain growth. Because 4Y-PSZ contains a substantial cubic fraction, transformation toughening is less pronounced than in 3Y-TZP; therefore, the possible mechanical consequences of excessive grain coarsening require direct evaluation [5,6]. Overall, extending the dwell beyond that needed for densification offered only marginal optical gains while promoting grain coarsening; a 1- to 2 h hold at 1530 °C therefore appeared sufficient for this material [5,23].

### 4.3. Effect of Heating Rate

In contrast to peak temperature and holding time, heating rate had no significant effect on final grain size within the tested range. This outcome can be understood from the kinetics of grain growth during sintering. Grain growth is a thermally activated, time-dependent process, and the great majority of grain-boundary migration occurs during the isothermal dwell at peak temperature rather than during the comparatively brief heating ramp. Within the tested range (3–10 °C/min), the difference in total time spent at high temperature during the ramp is small relative to the 120 min dwell, so the cumulative thermal exposure governing final grain size is dominated by the dwell rather than by ramp rate. This interpretation is consistent with the report of Öztürk and Çelik, who found no significant difference in the average grain size of monolithic zirconia across heating rates of up to 40 °C/min when peak temperature and holding time were held constant [25]. Heating rate can, in principle, influence microstructural evolution by altering the relative onset of densification and grain growth–slower heating allows more pore elimination before substantial grain coarsening begins, whereas very fast heating may trap pores or generate transient thermal gradients–but under the present conditions these effects were not large enough to produce significant differences in final grain size or defect density [24]. This suggests that, when peak temperature and dwell are held constant, the final microstructure is governed primarily by the dwell at peak temperature rather than by ramp rate. Heating rate showed only limited associations with transmittance, with a non-monotonic pattern. Although HR7 showed the highest model-predicted defect density and the lowest mean transmittance, no significant overall effect of heating rate on defect density was detected, and this pattern should therefore be interpreted with caution. The small specimen-level sample size for transmittance further limits the strength of these inferences. Overall, these data suggest that, within the tested range, ramp speed was a secondary factor compared with peak temperature and holding time. This finding broadly agrees with the systematic review and meta-analysis by Liu et al. [7]. That analysis found generally comparable optical and mechanical outcomes between speed-sintered and conventionally sintered zirconia, although some translucency parameters were reduced after speed sintering. Alshahrani et al. reported that speed- and high-speed sintering can compromise translucency in some Y-stabilized zirconia materials, underscoring material-dependent responses [29]. Other in vitro studies have reported comparable optical or mechanical properties after fast sintering in selected zirconia systems [18,19,20,23]. For example, Cokic et al. reported that speed sintering affected 3Y-TZP and 5Y-PSZ differently, further supporting material-specific responses [18].

The present findings extend this pattern to the tested 4Y-PSZ material, in which faster heating produced only small changes in transmittance under the evaluated conditions [7,18,19,20,23,30]. This relative stability may reflect the higher cubic fraction of 4Y-PSZ, which can reduce the sensitivity of optical properties to minor microstructural variations [5,6]. By contrast, 3Y-TZP may be more sensitive to accelerated sintering because of its stronger dependence on complete densification and birefringence-related scattering at tetragonal grain boundaries [5,6]. All tested schedules produced specimens with total luminous transmittance ≥40% at 0.5 mm thickness, suggesting that moderate acceleration may be feasible for this 4Y-PSZ material when near-full density is achieved.

### 4.4. Relationship Between Defect Density and Transmittance

A notable feature of this study was the quantitative assessment of area-normalized defect density alongside total luminous transmittance. The 180 min hold produced the lowest defect density and the highest measured transmittance, supporting the role of residual porosity in optical scattering [5,12,15]. In general, conditions that reduced defect density also tended to produce slightly higher transmittance values. However, transmittance is multifactorial and is not governed solely by porosity. Tp1560 illustrates this point: despite having the lowest defect density, it showed slightly lower transmittance than the 1470–1530 °C groups. This suggests that grain size and phase assemblage may counterbalance the optical benefit of reduced porosity. In 4Y-PSZ, the substantial cubic fraction may mitigate birefringence, but excessive grain growth may still increase scattering from retained tetragonal regions [5,6,12]. Therefore, an optimal microstructure for translucent 4Y-PSZ likely requires near-full density, controlled grain size, and a favorable phase assemblage [5,6,12]. Within the tested sintering window, a reasonable balance appeared to be achieved at 1500–1530 °C with a 1–2 h hold. Although mechanical properties were not directly measured, the quantified defect density may have mechanical relevance because internal pores can act as stress concentrators [5,13,14]. However, this possibility requires direct mechanical testing. Prior research suggests that insufficient sintering can increase porosity and reduce strength, whereas excessive grain growth may eventually dominate failure behavior [5,13,14].

In the present study, the 1560 °C condition showed the lowest defect density, but its enlarged grains may offset some benefits of reduced internal flaws [13]. The accelerated schedules tested here did not produce a substantial increase in defect density. This suggests that the tested 4Y-PSZ material may tolerate moderate acceleration without marked porosity-related microstructural compromise, although direct mechanical confirmation is needed [18,19,20,23]. Material-specific validation remains warranted for more aggressive protocols, such as total cycle times of less than 1 h or microwave-assisted sintering, because some studies have reported increased porosity or reduced translucency under such conditions [31,32]. Within the tested ranges, moderate acceleration did not substantially compromise microstructure or total luminous transmittance in this 4Y-PSZ material [5,7,18,19,20].

### 4.5. Integrated Effects of Sintering Parameters on Microstructure and Transmittance

Taken together, the three sintering parameters exerted distinct and hierarchical effects on the microstructure of monolithic 4Y-PSZ. Peak temperature exerted the strongest influence on grain size, which increased significantly and monotonically, and was associated with the lowest defect density at the highest temperature (1560 °C); notably, the only optical penalty also appeared at 1560 °C, where enlarged grains and phase-assemblage effects offset the benefit of reduced porosity. Holding time produced directionally similar effects—increasing grain size and, at the longest dwell (180 min), significantly reducing defect density and slightly increasing transmittance—reflecting the time-dependent nature of the same diffusion-controlled processes. Heating rate, by contrast, was a secondary factor that did not significantly alter final grain size or defect density within the tested range. Across all three parameters, total luminous transmittance varied within a narrow band (approximately 40–43% at 0.5 mm thickness), with only isolated significant pairwise differences, indicating that—once near-full density is achieved—the optical performance of this 4Y-PSZ is relatively insensitive to moderate variations in the firing schedule. This hierarchy (peak temperature > holding time > heating rate) provides a practical basis for prioritizing process control during clinical sintering.

### 4.6. Clinical Implications

The present findings help define practical sintering conditions for the tested high-translucency 4Y-PSZ material. To optimize transmittance while limiting unfavorable microstructural changes, the sintering schedule should promote near-full densification without excessive grain coarsening. The data suggest that a peak temperature of approximately 1500–1530 °C may be appropriate for this material, whereas 1560 °C produced diminishing optical returns and greater grain growth [5,13,14,15]. A holding time of 1–2 h at peak temperature appeared sufficient under the tested conditions; longer holds offered minimal optical benefit and promoted grain coarsening [5,23]. When faster turnaround is required, shorter holding times or faster heating rates may be feasible for this 4Y-PSZ material, but validation of mechanical performance and aging resistance is needed [7,18,19,20]. This interpretation is consistent with systematic reviews showing that speed-sintering effects vary by zirconia formulation and outcome measure [7,23]. Brand-specific responses to ultra-fast schedules have been reported, and differences may be more pronounced in 3Y-TZP than in 4Y- and 5Y-based materials [5,6,21].

Manufacturers’ guidelines and material-specific validation should therefore be used when deviating from conventional firing protocols. The quantitative defect analysis further indicates that sintering protocols leaving appreciable residual porosity may compromise optical quality. Although the observed transmittance differences may be small visually, internal pores may still affect long-term mechanical reliability as stress concentrators [5]. From a materials-science perspective, the objective is to achieve complete and uniform densification while controlling grain growth.

Future research should examine how the observed microstructural changes influence biaxial flexural strength, fracture toughness, hardness, and hydrothermal aging resistance in 4Y-PSZ. It would also be valuable to test whether the non-monotonic heating-rate pattern observed here extends to other 4Y-PSZ products or reflects specimen-specific variability. Overall, by isolating peak temperature, holding time, and heating rate, this study provides a quantitative framework for process–structure–optical property relationships in translucent 4Y-PSZ [5,6,7,23].

### 4.7. Limitations

Several limitations should be acknowledged. First, only a single commercial 4Y-PSZ product (Estar-Z HT; Osstem Implant Co., Ltd., Seoul, Republic of Korea) from one manufacturer was evaluated. Although this design minimized material-related confounders, the findings may not be directly generalizable to other 4Y-PSZ formulations or to zirconia ceramics of different compositions, such as 3Y-TZP or 5Y-PSZ. Future studies should include multiple commercial products to determine whether the identified sintering window is material-specific or broadly applicable.

Second, mechanical properties, including biaxial flexural strength, fracture toughness, hardness, and low-temperature degradation behavior, were not evaluated and warrant confirmation in follow-up studies. Third, defect-density analysis was based on two-dimensional polished cross-sections; three-dimensional porosity, which could be characterized by micro-computed tomography, was not quantified.

Fourth, color-related parameters, including CIELab- and CIEDE2000-based translucency parameters, contrast ratio, and CIE ΔE, were not measured; future studies incorporating these parameters would further characterize the clinical color behavior of the material.

Fifth, although each parameter series was analyzed independently and multiple comparisons were corrected within each series, no formal correction was applied across the three series and three outcomes; therefore, the family-wise Type I error rate across the entire study may exceed the nominal α = 0.05.

Sixth, the reference condition (1530 °C/120 min/5 °C/min) was prepared separately for each parameter series. Absolute values differed among these reference groups across series, indicating run-to-run variability in the sintering furnace. Consequently, cross-series comparisons should not be overinterpreted, and effects were evaluated only within each series.

Seventh, the specimens used for transmittance measurement were 0.5 mm thick, which is thinner than the ≥1 mm typically encountered in clinical monolithic restorations. Because total luminous transmittance is thickness-dependent, the absolute values reported here are not directly transferable to clinical thicknesses and should be interpreted on a relative, between-group basis. Specimen thickness may also influence heat transfer and thermal gradients during sintering; thinner disks could reach thermal equilibrium more readily than thicker clinical geometries, so the densification behavior observed here may not fully replicate that of clinically dimensioned restorations.

Eighth, separate specimens were used for optical measurement (*n* = 3) and for microstructural (SEM) examination (*n* = 3), because the SEM specimen preparation—including sectioning, polishing, and thermal etching—is destructive and precludes subsequent transmittance measurement on the same disks. In principle, transmittance could have been measured first on all six specimens before allocating them to destructive analysis, which would have increased the effective sample size for the optical measurements; this was recognized as an experimental limitation. The resulting small sample size for transmittance (*n* = 3 per group) limits the statistical power of the Kruskal–Wallis and Dunn’s pairwise comparisons, and the observed differences, particularly those with borderline *p*-values, should be interpreted with caution.

## 5. Conclusions

Within the limitations of this in vitro study, the following conclusions can be drawn for the tested monolithic 4Y-PSZ material (Estar-Z HT).

1. Higher peak sintering temperatures and longer holding times increased grain size and reduced internal defect density. These microstructural changes produced only modest changes in total luminous transmittance, indicating diminishing optical returns once near-full density is approached.
2. Variations in heating rate (3–10 °C/min) had no significant effect on final grain size or defect density and only limited associations with transmittance.
3. All sintering schedules yielded broadly comparable total luminous transmittance values of approximately 40–43% at 0.5 mm thickness. Thus, the tested monolithic 4Y-PSZ material showed minimal optical penalty under moderately accelerated sintering protocols, provided that over-sintering at 1560 °C was avoided.
4. A peak temperature of 1500–1530 °C combined with a holding time of 1–2 h appeared to provide a favorable balance between densification, grain coarsening, and optical transmittance for the tested monolithic 4Y-PSZ material.
5. Before broader clinical recommendations can be made, this processing window should be validated through mechanical testing (biaxial flexural strength, fracture toughness, and hardness), low-temperature (hydrothermal) aging studies, and evaluation of additional commercial 4Y-PSZ products.
6. By independently isolating peak temperature, holding time, and heating rate, this study provides a quantitative process-structure-property framework that can guide the optimization of sintering protocols for high translucency 4Y-PSZ. These findings are directly relevant to dental CAD/CAM workflows, where reliable translucency and efficient furnace scheduling are both clinically important, and the quantitative defect-density approach may also inform the broader processing of translucent zirconia ceramics for biomedical and optical applications.

## Figures and Tables

**Table 1:** Sintering schedules for experimental groups.

Group	Peak Temperature (°C)	Holding Time (min)	Heating Rate (°C/min)
Peak-temperature series			
Tp1470	1470	120	5
Tp1500	1500		
Tp1530 (reference)	1530		
Tp1560	1560		
Holding-time series			
Tm30	1530	30	5
Tm60		60	
Tm120 (reference)		120	
Tm180		180	
Heating-rate series			
HR3	1530	120	3
HR5 (reference)			5
HR7			7
HR10			10

Footnote: Tp1530, Tm120, and HR5 correspond to the same nominal sintering condition (1530 °C/120 min/5 °C/min), which served as the reference in each parameter series; specimens for each series were prepared in independent furnace runs.

**Table 2:** Mean grain size of 4Y-PSZ under different sintering conditions.

Sintering Parameter	Group	Grain Size (μm)
Peak temperature	Tp1470	0.481 ± 0.020 ^d^
	Tp1500	0.569 ± 0.030 ^c^
	Tp1530	0.671 ± 0.036 ^b^
	Tp1560	0.785 ± 0.035 ^a^
Holding time	Tm30	0.503 ± 0.037 ^d^
	Tm60	0.579 ± 0.020 ^c^
	Tm120	0.669 ± 0.027 ^b^
	Tm180	0.730 ± 0.041 ^a^
Heating rate	HR3	0.701 ± 0.032 ^a^
	HR5	0.708 ± 0.024 ^a^
	HR7	0.721 ± 0.034 ^a^
	HR10	0.727 ± 0.068 ^a^

**Footnote:** Values are mean ± standard deviation (SD) based on *n* = 10 fields per group (≥100 grains per group in total). Mean grain size was calculated using the linear-intercept method in accordance with ASTM E112-24 and Mendelson’s correction (d = 1.56 × L^−^). Within each parameter series, values with different superscript letters differ significantly (*p* < 0.05; one-way ANOVA with Tukey’s HSD test for the peak-temperature and holding-time series and Welch’s ANOVA with the Games–Howell test for the heating-rate series). In the heating-rate series, the shared superscript “a” indicates that no pairwise comparison reached statistical significance.

**Table 3:** Model-predicted internal defect density, defined as defects ≥0.5 μm, of 4Y-PSZ under different sintering conditions.

Sintering Parameter	Group	Predicted Defect Density (counts·mm^−2^)	95% CI
Peak temperature	Tp1470	84.44 ^b^	58.68–121.50
	Tp1500	96.08 ^b^	68.31–135.15
	Tp1530	55.32 ^b^	35.29–86.73
	Tp1560	23.29 ^a^	11.65–46.58
Holding time	Tm30	52.41 ^b^	33.02–83.18
	Tm60	37.85 ^b^	21.98–65.19
	Tm120	32.03 ^b^	17.74–57.83
	Tm180	8.73 ^a^	2.82–27.08
Heating rate	HR3	116.46 ^a^	85.43–158.77
	HR5	131.02 ^a^	97.82–175.48
	HR7	192.16 ^a^	150.97–244.59
	HR10	157.22 ^a^	120.42–205.28

**Footnote:** Values are model-predicted mean defect densities with 95% confidence intervals (CIs) obtained from a Poisson generalized linear model with a log link and log-transformed analyzed area as an offset. *n* = 30 fields per group. Within each parameter series, values with different superscript letters indicate significant pairwise differences (*p* < 0.05), with pairwise contrasts performed using the most-densified group as the baseline. Lower values indicate more complete densification.

**Table 4:** Total luminous transmittance of 0.5 mm-thick 4Y-PSZ disks under different sintering conditions.

Sintering Parameter	Group	Transmittance (%) (Mean ± SD)
Peak temperature	Tp1470	42.37 ± 0.04 ^a^
	Tp1500	42.28 ± 0.02 ^ab^
	Tp1530	42.37 ± 0.02 ^ab^
	Tp1560	41.66 ± 0.02 ^b^
Holding time	Tm30	42.92 ± 0.03 ^b^
	Tm60	43.05 ± 0.02 ^ab^
	Tm120	43.16 ± 0.02 ^ab^
	Tm180	43.35 ± 0.03 ^a^
Heating rate	HR3	41.92 ± 0.06 ^a^
	HR5	41.86 ± 0.02 ^ab^
	HR7	40.79 ± 0.07 ^b^
	HR10	40.90 ± 0.03 ^ab^

Footnote: Values are group means ± standard deviation (SD) based on *n* = 3 specimens per group; each specimen was measured three times, and the average value for each specimen was used for analysis. Measurements were performed in accordance with ISO 13468-1:2019 under CIE standard illuminant D65 on double-sided mirror-polished disks (13 mm diameter × 0.5 mm thickness). Within each parameter series, values with different superscript letters differ significantly (*p* < 0.05; Kruskal–Wallis test followed by Dunn’s pairwise comparisons with Bonferroni correction). Higher values indicate greater transmittance.

## Data Availability

The datasets generated during and/or analyzed during the current study are available from the corresponding author on reasonable request.

## Abbreviations

The following abbreviations are used in this manuscript:

4Y-PSZ	4 mol% yttria-partially stabilized zirconia
3Y-TZP	3 mol% yttria-stabilized tetragonal zirconia polycrystal
CAD/CAM	Computer-aided design/computer-aided manufacturing
LTD	Low-temperature degradation
SEM	Scanning electron microscopy
ASTM	American Society for Testing and Materials
ANOVA	Analysis of variance
HSD	Honestly significant difference
LRT	Likelihood-ratio test
IRR	Incidence rate ratio
CI	Confidence interval
SD	Standard deviation
CIE	Commission Internationale de l’Éclairage
CIELab	CIE L*a*b* color space
CIEDE2000	CIEDE2000 color-difference formula
ISO	International Organization for Standardization
SPSS	Statistical Package for the Social Sciences
